# THE GENERALIST RESOURCE TO INTEGRATE DRIVING: MEASURING THE CLINICAL UTILITY OF THE GRID TOOL

**DOI:** 10.1093/geroni/igae098.2576

**Published:** 2024-12-31

**Authors:** Anne Dickerson, Terri Cassidy, Susan Touchinsky

**Affiliations:** East Carolina University, Greenville, North Carolina, United States; Fitness to Drive, Colorado Springs, Colorado, United States; Adaptive Mobility Services, LLC, Orwigsburg, Pennsylvania, United States

## Abstract

Cut-off scores for driving evaluations are based on the concept that a score indicates whether the individual is likely to pass or fail a driving evaluation. Cut-off scores should be derived from research over multiple studies used with diverse populations. Assessments develop scores based on specific populations (e.g., dementia) which may not be valid for other populations (e.g., stroke). Thus, when using scores, it is critical to understand the population and range of scores upon which it is based. The Generalist’s Resource to Integrate Driving (GRID) is a resource for practitioners to integrate driving into their practice and use scores appropriately. The tool is designed to provide specific guidelines to evaluate, create and implement intervention plans using the schematic of red (stop driving), green (ok to drive) and yellow (needs further evaluation). The aim of this study was to evaluate the utility of the GRID by practitioners who used it. Results of the IRB approved Qualtrics survey with 70 respondents showed 79% indicated they used while 21% had not, although 10% plan to use it. Also, 69% of users found it easy-to-use, 73% indicated it was easy to understand, and 63% felt it was comprehensive. and use scores appropriately 51% respondents indicated the most important use was screening for driving risk although planning a referral to a driving evaluation was a close second. This presentation will briefly explain the use of the GRID, the model upon which it is based and discuss the results of the utility study.

